# COVID‐19 as a breakdown in the texture of social practices

**DOI:** 10.1111/gwao.12524

**Published:** 2020-09-29

**Authors:** Michela Cozza, Silvia Gherardi, Valeria Graziano, Janet Johansson, Mathilde Mondon‐Navazo, Annalisa Murgia, Kim Trogal

**Affiliations:** ^1^ Department of Organization and Management, School of Business, Society and Engineering Mälardalen University; ^2^ Research Unit on Communication, Organizational Learning, and Aesthetics University of Trento; ^3^ Faculty Research Centre in Postdigital Cultures Coventry University; ^4^ Department of Management and Engineering, Business Administration Linköping University; ^5^ Department of Social and Political Sciences University of Milan; ^6^ Canterbury School of Architecture University for the Creative Arts

**Keywords:** care, diffraction, invisibility, mending, repair

## Abstract

‘A lot of things need to be repaired and a lot of relationships are in need of a knowledgeable mending. Can we start to talk/write about them?’ This invitation — sent by one of the authors to the others — led us, as feminist women in academia, to join together in an experimental writing about the effects of COVID‐19 on daily social practices and on potential (and innovative) ways for repairing work in different fields of social organization. By diffractively intertwining our embodied experiences of becoming together‐with Others, we foreground a multiplicity of repair (care) practices COVID‐19 is making visible. Echoing one another, we take a stand and say that we need to prevent the future from becoming the past. We are not going back to the past; our society has already changed and there is a need to cope with innovation and repairing practices that do not reproduce the past.

## 1 INTRODUCTION

**Silvia Gherardi:** The story of this ‘experimental writing’ began with an email that I sent to some colleagues and friends with whom I was already in contact or interested to enter into correspondence with. The invitation to write collaboratively for the section *Feminist Frontiers* was formulated in the following words:

*In the past I developed a theoretical framework for studying workplace accidents as breakdowns and to study mending practices for the repair work of the texture of practices (Gherardi,* *2004* *)*.

*At present I would like to invite other scholars who have or have not yet reflected on ‘repair work’ and engage them in an experiment in collaborative writing about the effects of Covid‐19 on daily social practices and on potential (and innovative) ways for repairing work in any possible field of the social organization. To give the flavor of what I wrote about mending the social texture, I extract few lines from that article:*

‘Repair has been studied mainly by ethnomethodology (Schegloff, 1992; Sudnow, 1978), which considers repair to be a technique used to fix breakdowns in language and in conversation. When ethnomethodology and studies of practice are considered together — as in Henke (2000) — social and material forms of order become closely integrated. Henke proposes a sociology of repair to analyse it as an ongoing skill used to maintain workplace order. He writes: “repair is not at the margins of order, waiting to be deployed if something goes wrong. Instead it is a practice at the centre of social order: repair work makes workplaces normal” (Henke, 2000, p. 55). Whereas Henke studies repair technicians in order to show that repair work involves more than working on machines and other material artefacts, and that repair workers also fix the social order.’*Recently, Covid‐19 has made me think that repairing the social order is not only a sensemaking activity but a social practice of engineering the heterogeneous. Ethnomethodologists use the term ‘remedial’ or ‘repair’ (Goffman,* *1971* *; Owen,* *1983* *) in relation to the practice of mending the social order. I prefer to use the term ‘mending’, or its synonym ‘darning’ for two reasons: firstly, to link it with the metaphor of texture, and secondly to emphasise the ‘gendered’ character of mending work. Darning, in fact, relates more to the domestic sphere, to a manual skill that is disappearing, and which has never been noble or extolled in its social representation. The femaleness associated with darning is intended to connote it as a humble activity, one that is not seen or only noted by its absence, like the majority of domestic ancillary and support activities*.

*Language is important because it is a means of ‘worlding’. The language of Covid/pandemic is often formulated in terms of war and related metaphors (health personnel as ‘heroes’). Can the language of care and response‐ability be an antidote that deploys a different imaginary?*

*In relation to breakdowns and heterogeneous engineering, a pandemic may be defined, from a sociological perspective, as a breakdown in the social fabric. Thus, the processes of repair and reconstruction of the ordering modes of so‐called ‘normalcy’ may be seen as mending activity. The fundamental processes of repair and reconstruction of social order after a breakdown represent a process of engineering heterogeneous elements*.

*I used the metaphor of the texture of organizing (Gherardi & Strati,* *1990* *) to describe how the normalcy of organizational life (and of society at large) is the product emerging from relations of connectedness in action, how this connective texture is taken‐for‐granted when the alignment of ideas, persons, materials and technologies holds together, and how this texture is subject to rips and tears, which require skilful mending work for their repair. Can we use the concepts of texture, breakdown and mending in thinking about the effects induced by Covid‐19?*

*The conceptualization of breakdowns in sociomaterial terms erases the distinction between the social and the technical. In this case, the boundaries among the sanitary, technical, human, political, managerial, psychological or cultural vanish, so that a pandemic can be defined simply as a breakdown or dis‐alignment in what hitherto was a way of ordering heterogeneous materials. We are writing at the beginning of Phase 2, but it is evident that it cannot just be a return to good old times. Covid‐19 has exacerbated the old inequalities and created new ones. Could it become an opportunity for learning and innovating? And if so, where, how, and by whom?*

*In Phase 2 the texture of connections is emerging from dispersed and fragmented actions, and the more the action is held together by a mode of ordering the heterogeneity of people, things and ideas, the more it is taken for granted. Yet it is fundamentally fragile and subject to constant negotiation and renegotiation by those involved in weaving its threads together. The pandemic is a breakdown in what was previously taken for granted, and in the situation of normalcy that had been patiently woven together in the micro‐interactions of everyday life. This breakdown upsets the emotional as well as social order, and those of decisions and actions. Consequently, disorder takes over from order, and actors find themselves wittingly or unwittingly engaged in normalcy repair practices. As the damaged texture of normalcy is mended, reflexivity may arise about practices that were previously taken for granted, so that the pandemic may provide an opportunity for discussion of the previous social order and the introduction of change*.

*Can we start a conversation about mending, having in mind a feminist ethos of care?*

*I put forward my work on repair work to make explicit my concern about ‘what happens next after the lockdown’. Moreover, I wish to mention a special issue, edited by Graziano and Trogal (* *2019* *) (entitled Repair Matters) that explores the politics of repair in these terms:*

‘In order to explore the politics of repair in the context of organization studies, we focus on four aspects of reflection that we believe will become central to further discussion in the coming political phase: 1) repair as a specific kind of labour of care and social reproduction; 2) repair as a direct intervention into the cornerstones of capitalist economy, such as exchange versus use value, work regimes and property relations; 3) repair of our material world and logistical infrastructures; and finally 4) the repair of our immaterial world, including the ways in which we think about complex systems and institutional practices.’ *(Graziano & Trogal,* *2019* *, p. 204)*

The invitation letter had a final comment: ‘a lot of things need to be repaired and a lot of relationships are in need of a knowledgeable mending. Can we start to talk/write about them?’ I sent this kind of invitation to Valeria Graziano who in her turn extended it to Kim Trogal; Michela Cozza; Annalisa Murgia, who extended it to Mathilde Mondon‐Navazo; Janet Johansson and one more person who could not accept due to being overwhelmed by current engagements. While writing we were living our #IStayHome in different countries and under different legal restrictions on our freedom: Valeria in Rijeka, Croatia and Kim in London, Michela and Janet in Sweden (respectively in Västerås and in Stockholm), Mathilde in Paris, Annalisa in Milan and Silvia in Trento.

In what follows the reader may have the feeling of how our dialogue unfolded and how we developed a prismatic vision on breakdown, repair and care. The different contributions are stitched together: every section ends by introducing the following one in an effort to reproduce the becoming of the dialogue among us.

The reader is introduced to such collective writing by Michela’s concern about the in‐visibility of the gendered healthcare work. These thoughts become Silvia’s reflection on (in)visibilization as an effect of COVID‐19. Annalisa and Mathilde build on Silvia’s words to discuss the situation of platform riders during the pandemic. In turn, this case brings Valeria and Kim to elaborate on the inequalities of labour at the time of COVID‐19. This caring for the unequal impacts of production during the crisis turns into Janet’s invitation to embrace the inevitable change imposed by the pandemic and to care for developing a new vision of our existence in connection with others and nature. The final collective epilogue further sews together our thoughts within a feminist texture, which positions us in dialogue with the many authors of this *Feminist Frontiers* section.

## 2 THE IN‐VISIBILITY OF GENDERED HEALTHCARE WORK AT THE TIME OF COVID‐19

**Michela Cozza:** Inspired by Silvia’s invitation to reflect upon the relation between the pandemic and repair work, I was the first to send back a reflection that points out how COVID‐19 is making healthcare work visible, which is among the most invisible gendered work on which the ‘repair and maintenance’ of society depends.

Repair takes place when a breakdown has occurred; similarly, a pandemic can be described as a global breakdown that cuts across social, organizational and technical/technological components, and needs both prompt intervention and coordinated practices to bring back human bodies, organizations and sociotechnical infrastructures to their lives and businesses. From a feminist perspective, repair can be understood as a subset of care practices (Graziano & Trogal, 2019), and just as the repair work is relevant to social and productive activities, care practices are key to health and wellbeing. However, both repairing in broad terms and caring as a particular kind of repair work are usually taken for granted and only a disruptive event foregrounds their instrumentality.

These days, while the coronavirus pandemic makes visible the importance of repairing functions embedded in healthcare work, the gendered feature of this work is still pretty invisible. Pandemics are rarely addressed from the point of view of those who are required to enact pandemic response procedures on behalf of governments and their publics, as happened with regards to the 2009 H1N1 pandemic (Davis, Flowers, & Stephenson, 2014). It is no surprise that gender has not been addressed in either outbreak responses and policies — ‘characterized by the “tyranny of the urgent”, which puts aside structural issues in favour of addressing immediate biomedical needs’ (Smith, 2019, p. 357) — as well as in research, as occurred at the times of the Ebola and Zika outbreaks that, however, highlighted the need to address gender issues relating to the spread and control of infectious diseases.

Pandemics — coronavirus being no exception — give prominence to the vital importance of healthcare workers who strive to ‘repair’ bodies whose functioning has been compromised and, quite often, they also care by comforting patients, as many nurses tell on various platforms that collect their COVID experiences in the form of public journals, YouTube interviews and podcast stories. An excerpt from a nurse’s story follows:^1^

*The 80‐year‐old female looks frightened as I enter. The HEPA filter drones loudly in the corner of the room. I have to shout through my mask to be heard as I ask her how she’s doing and if she feels short of breath. She is comfortable but afraid*. I sense her loneliness and stroke her hair gently *and then take her blood glucose. I listen to her rhonchorous and rattly lungs with a portable stethoscope and then leave her room to get her tray*.

It is a heavy emotional burden that, in some cases, mean that nurses are in need of being mentally and physically ‘repaired’ in their turn. ‘I realize that the *mental anxiety* that has been running through my veins for weeks on end since we’ve been talking about this *is physically making me sick*’ says another nurse.^2^ Despite being aware of their own vulnerability, many healthcare workers acknowledge that they are part of a very peculiar scene where they are asked to play a role that places them at risk. However, others’ needs have primacy, as another nurse tells us:^3^

*I will not tell you about the sacrifices that each of us makes, the fear we feel, the very sad episodes we are witnessing. Situations are such that sometimes tears burn our own eyes but we hold back as much as possible so as we do not break down those who would have more rights than us, that is, the patients who live these situations in first person and who hold onto our smiles hidden behind the masks*.

*(Original in Italian:* Non vi racconto i sacrifici che ognuno di noi fa, la paura che proviamo, gli episodi tristissimi a cui assistiamo. Situazioni tali che a volte ti bruciano gli occhi dal pianto ma ti trattieni il più possibile per non far crollare chi più di te ne avrebbe diritto, ovvero i malati che vivono queste situazioni in prima persona e che si aggrappano ai nostri sorrisi celati dietro alle mascherine.*)*

Healthcare workers — mainly women — are expected to put themselves at great personal risk for the community but, as occurred during past pandemics, governments and global health institutions are paying scant or even no attention to the gendered impacts of the outbreak (Wenham, Smith, & Morgan, 2020).

People in different countries are applauding their medical staff as tireless heroes but such a heroic visibility is gender neutral and, in some countries more than others, its racial dimension is also silenced. Once again, the western‐white‐masculinity is the universal. Again, history repeats itself. In fact, in 1918, on the occasion of the influenza pandemic in Canada:

*[n]ewspaper reports acknowledged the efforts and skills of male doctors and politicians, claiming that their knowledge and expertise proved crucial in guiding the city in the face of chaos. Yet the archival record shows that it was often highly trained, senior female nurses who were central to the management of emergency influenza services*. (Godderis & Rossiter, 2013, p. 306)

At the time of COVID‐19, the same gender‐blindness is manifesting. I wonder how many new pandemics must strike before gender is made visible in healthcare work … before caring for front‐line healthcare providers … before interrogating the definition of what counts as work.

*Such a concern about gender‐blindness and the feminine in care work prompted a dialogue on the making of visibility/invisibility*.

## 3 (IN)VISIBILIZATION AS COVID‐19’S EFFECT

**Silvia Gherardi:** Everything seems to have been said and written about the impact of the coronavirus on our contemporary society and I wonder if there really is still something to be said that has not already been said. As I continue to ask myself this question and I feel discomfort and suffering growing in me, I think there is still a need to say that COVID‐19 has produced a rarefaction of the social fabric that remains hidden. But at the same time, COVID‐19 has made visible how the social fabric has become sparse and vulnerable.

There is a symptom in the language that denotes the way through which the collective unconscious manifests itself. The term social distancing is widely used and apparently seems to be understood by everyone to indicate the physical distance that is suggested between one person and another. But this is precisely the symptom since physical distancing is taken as synonym of social distancing. While a physical distance is easily visible and maybe even controlled by a drone, what remains invisible is the social distancing and the suffering it produces. We need social proximity while maintaining physical distance. In fact, the health, economic and political crises prompted by COVID‐19 have entered the visibility regime but the social crisis understood as the suffering produced by the absence of forms of ordinary sociality belongs to the realm of invisibility.

The hashtag #IStayHome does not have the same meaning for everyone, rather it covers the pandemic inequalities that the rarefaction of the social fabric is producing. It is trivial to emphasize that not everyone has a home, or that inequalities in the forms of living accentuate the discomfort with which the individual and families are at home. I do not want to mention extreme forms of vulnerability that have been accentuated by the pandemic — not only those who live on the street, but also those whose confinement is in jail, elderly nursing homes or refugee camps. For them, #IStayHome is simply paradoxical or just an insult. I have in mind the ordinary middle‐class family for whom confinement is synonymous with containment and ultimately with freedom.

Therefore, it is better to speak of forced domesticity if we want to make visible how gender, race and class contribute to making the social fabric diverse. The support network that social services, the family, neighbourhood and friendships that in ‘normal’ times could remedy the many frailties both individually and in a family context, is now broken. Forced domesticity often has different gender effects.

New words appear in the Italian vocabulary that prefer to keep the English expressions in use (in a more or less correct form). This is how ‘smart working’ and ‘home schooling’ made their appearance in Italian. What remains in the shadows is how it is possible to do smart work and take care of your children at the same time, and who in the family takes care of them. What is not acceptable is that the public discourse presents ‘smart work’ as especially suited for moms while schools and other children’s services are closed, and it is even presented as money‐saving in place of hiring a babysitter. What stays in the shadows is the gendered effect produced by a reduction in income that entails an intensification of work in the kitchen and of the time for cooking less expensive food while maintaining a balance between health and domestic economics. Forced domesticity is neither identified with creativity in the kitchen, at least not for everyone, nor with meals ordered online at restaurants and brought home by the workforce that in these days has sewn and repaired, through the market, many forms of sociality made impossible by the lockdown.

Social services have been frozen, and the family forced to make up for both the service, discomfort and suffering that this entails. Services for the elderly such as day centres have been shut, closed schools are depriving a generation of a constitutional right to education, children in need of support are kept at home. The social uneasiness produced by forced domesticity has been partially mitigated by various forms of solidarity and by courageous and voluntary initiatives by individuals and associations that have been mobilized. The anti‐violence centres have been reorganized in many territorial realities, thus making the effect of forced domesticity visible.

Some territories — where the establishment of institutions in society was already a consolidated fact and where several small municipalities were connected, for example, to share services — have shown considerable resilience. I have in mind the Emilia‐Romagna region where some unions of municipalities have experimented with unexpected forms of activating services.^4^ This was possible thanks to the initiative of the mayors of the small municipalities who took responsibility for inventing creative responses to the old and new social needs present in the families of the territory.

#IStayHome encompasses the romantic aura of an unprecedented and individually challenging experience and a legal obligation and civic duty. The three flavours mix and give rise to rhetoric and ambivalent forms of social behaviour. The individual commitment to look for forms of coping to resist isolation and anxiety has challenged the imagination of the individual who uses Zoom to organize aperitifs and dinners in the virtual company of friends and relatives. On the balconies of many houses drawings of rainbows appeared and the words ‘everything will be fine’; people came out on their balconies to sing and Italy found itself united for 25 April to rediscover and sing at the top of their lungs ‘Bella Ciao’. But #IStayHome’s obligation was also expressed with social control and reporting to the police of those who went out for walks, even on country roads, or with insults from the balcony to those on bicycles. The civic virtue of those who stayed at home for the good of all and the state did not dissociate themselves from social control and sadistic pleasure for their virtue. The principle that the more one withdraws into the private, the more this benefits the public, redesigns the boundaries between public and private. With the forced domesticity the public relegates the private with its own problems, difficulties and concerns. While forced domesticity has traditionally been the condition for people with poor health conditions, disabilities or legal problems, now it is the condition for almost everybody, even if its democratic image is only apparent while it hides the vulnerabilities of women, the elderly and children.

What is visible is the prohibition to stay in public spaces and those who do so receive more visibility. This visibility is not democratic. The visibility of healthcare professionals was emphasized by the rhetoric of heroism and the care of others that required self‐denial. The invisibility instead accompanied the intensification of the traffic of couriers who delivered all types of goods and supported the COVID‐economy. The same invisibility has covered the domestic and industrial cleaning industry, as well as the care of carers and agricultural work, sectors where gender and race are inscribed in bodies and care jobs.

A few days ago, at the suggestion of a dear friend, I was leafing through the latest book by Françoise Vergés (2019), *Un féminisme décolonial*, and I was reflecting on how the dynamics of visibility and invisibility are central to the theorization of postcolonial feminism. Historically, the subalterns, the oppressed, have moved between visibility and invisibility as a deliberate strategy. Through the interruption of the hegemonic narrative, its structural function of erasure and going into hiding is revealed as another deliberate choice of repairing the effects of visibility. While the signs of discrimination, like skin colour or gender, mark visibility as an instrument of exclusion, systematic invisibilities aim to hide real human suffering. In an interview by Gerber (2020) with Françoise Vergés, she mentions how

*visibility is also an element of capitalistic logic: things must be made visible to become objects and merchandise. Just look at the advertisement industry: everything — narratives, crafts, memories, etc. — must be packaged visually to enter the market, must fit the Instagram, TikTok, Twitter frames*.

In this pandemic period, I feel an urgent need to challenge the frames that certain ways of making events visible impose on our relations with the world and the others in this world. Decolonial feminism is a way of theorizing and practising a positioning in the world that acknowledges the contribution of the global South and fights against the appropriation of women’s rights by the states (what has been called femonationalism) and its institutionalization pursued by white feminism. Françoise Vergés argues that the feminism of western civilization has made the rights of women an ideology of assimilation and integration into the neoliberal order, ignoring the structural violence against racialized people. Decolonial feminism talks to me in relation to the growing of inequalities as an effect of the pandemic to remind us of the need to change our western epistemological assumptions and challenge a Eurocentric knowledge that is fragmented and hierarchical. COVID‐19 makes us prisoners of multiple temporalities: the past (and not only of colonialism), which is far from being repaired; the present, in which a breakdown has changed our forms of sociality; and the need to prevent the future from becoming the past. We are not going back to the past; our society has already changed and there is a need to cope with innovation and repairing practices that do not reproduce the past.

I wish to finish with an experience that marked me deeply. Yesterday evening I took part in a commemoration ceremony for a colleague who had passed away. It was held by Zoom and 52 persons attended. It was very moving, and it was a new form of digital mourning. I feel that in the present rarefaction of social bounds there is a need to grieve for those mothers that have given birth alone, those who have died alone and those who could not go to the cemetery to mourn their loved ones. The sociality of birth and death has been disrupted by physical distancing. The breakdown of ordinary sociality is going to mark the contemporary meaning of what it means to be a human being and becoming with the world.

*Silvia points out that ‘forced domesticity’ is not accessible to everyone as in the case of specific categories of workers who are denied that right*.

## 4 THE SITUATION OF PLATFORM RIDERS IN PARIS IN PANDEMIC TIMES^5^

**Annalisa Murgia and Mathilde Mondon‐Navazo:** In France as in many other countries, the lockdown was instigated to try to avoid the saturation of hospitals weakened by decades of spending cuts. In the resultant empty streets of Paris, one could see many platform deliverers riding their bicycles or motor scooters with their branded bags. But despite their undeniable presence in deserted public spaces, these riders were as invisible as usual.

On 1 April, Deliveroo — one of the main platforms organizing food delivery by self‐employed riders — released a short video on its social networks with the hashtag #ToujoursLàPourVousLivrer (*StillHereToDeliverToYou*).^6^ In one minute, Deliveroo wanted to underline its crucial role in the pandemic context, providing ready‐to‐eat meals to teleworkers in charge of their children or delivering free dishes to healthcare workers. According to this video, the first commitment of Deliveroo consists of supporting restaurants by allowing them to reduce their economic losses, as underlined by the final written sentence: ‘Deliveroo thanks and supports all restaurants in France’ (original in French: ‘*Deliveroo remercie et soutient tous les restaurants en France*’).

The *Collectif des Livreures Autonomes de Paris* (CLAP) — a grassroot group of platform riders — reacted with the hashtag #JamaisLàPourLesLivreurs (*NeverHereForTheRiders*)^7^ and the corresponding sentence: ‘Deliveroo exploits and despises all deliverers of France’ (original in French: ‘*Deliveroo exploite et méprise tous les livreurs de France*’). Indeed, while the video shows many restaurant owners and expresses a form of gratitude towards them, no single rider appears on screen, denying the role played by these workers in the delivery process.

Before the pandemic, several researchers had analysed the precarious situation of these unprotected self‐employed workers, who have poor working conditions and continuously decreasing remuneration (Bouvier, 2018; Jan, 2018; Leonardi, Murgia, Briziarelli, & Armano, 2019; Tassinari & Maccarrone, 2019). But their situation has become even worse in the context of the lockdown, as underlined by many riders from the Paris region.^8^ Nevertheless, despite their high exposure to contamination risks, platform deliverers do not seem to raise the same empathy and gratefulness granted to other workers, from healthcare to supermarket workers. Vincent explains: ‘There are at least ten contact zones for each deliverer in each building. So, it is very dangerous to enter the buildings, we need protections that we don’t have’ (original in French: ‘*Il y a au moins dix zones de contact pour un livreur dans chaque immeuble. Donc c’est très dangereux de rentrer dans les immeubles, il faut avoir des protections que nous n’avons pas*’).

The lockdown also resulted in frequent police controls to check the mobility certificates of people circulating in the streets. Vincent underlines that many riders are irregular migrant workers, who therefore have to choose between renouncing work or risking being arrested and held for a long period in administrative detention centres — where the health risk is very high — before being sent to their country of origin. Despite these risks, most riders do not have the possibility to withdraw, as they would not receive any compensation from the platforms they are working for. Hamza regrets:

*I would have liked to listen to the President of the Republic and be confined with my wife who is pregnant, but unfortunately I can’t.… If we don’t go to work tomorrow, we won’t have money to feed our family (original in French:* J’aurais aimé moi écouter le président de la République et être confiné avec ma femme qui est enceinte, mais malheureusement je ne peux pas.… Si demain on ne va pas travailler, on n’aura pas d’argent pour nourrir notre famille*)*.

But even putting their health at risk to go on working, riders are not able to earn enough money to make their living. With the closure of many restaurants, demand has dropped sharply, resulting in a decrease of about 33 per cent of hourly turnover according to Vincent. Deliveroo also used the pandemic context to remove their scheduling system, which used to regulate the number of online riders according to the expected order volume. Indeed, since 27 March, deliverers in the Paris region could connect themselves to the platform whenever they wanted, which increases the probability to work long hours without receiving any orders and consequently without being paid. And this change was introduced during a period when riders do not even have the possibility to gather and demonstrate to defend their rights. Riders are thus experiencing strained work conditions to carry on an activity that they do not perceive as socially meaningful or necessary. Vincent reports (translated in English):

*We are told how important riders are to keep delivering people, honestly, I have my doubts. We deliver kebabs, burgers, tacos, I am not sure it is vital. This is the first time I deliver so many ice creams since I started working.… I also delivered an order for a single bottle of wine: I received the order at Pigalle and delivered it at St‐Ouen [a four‐kilometre ride], at 400 metres from a supermarket.… I also delivered a box of biscuits that weighed 140 grams. (Original in French:* On nous dit que les livreurs sont importants pour continuer à livrer les gens, franchement j’ai des doutes. Nous on livre des kebabs, des burgers, des tacos, alors vital je sais pas. Depuis que je livre, c’est la première fois que je livre autant de glaces.… J’ai livré aussi une commande d’une seule bouteille de vin: j’ai réceptionné la commande au niveau de Pigalle pour la livrer à St‐Ouen, à moins de 400 mètres d’une superette.… J’ai livré aussi un paquet de gateaux qui faisait 140 grammes*.)*

There is thus a huge gap between the solidarity discourse held by platforms to demonstrate their social utility and the actual use of their services to satisfy frivolous cravings. As Hamza underlines: ‘It is a recreational service.… It is for people’s leisure and pleasure’ (original in French: ‘*C’est un service récréatif.… C’est pour le loisir, le plaisir des gens*’).

With the disdain expressed by delivery platforms towards their riders therefore comes a form of social contempt from socially privileged clients, who want to embellish their experience of the lockdown without considering the workers they are putting at risk. Not coincidentally, these ‘disposable workers’ — as Jérôme, a co‐founder of the CLAP, calls them — are mostly people of colour, that is, young men of African or Arab descent, as well as from an Asian background, living in popular neighbourhoods strongly impacted by the virus and subjected to police violence. If the coronavirus did not create these structural invisibilization dynamics based on class and race, it has sharply reinforced them, widening the gap between those forced into domesticity and those forced out, but also between those being applauded and those being forgotten. Hamza summarizes: ‘Of course we are on the front lines, but nobody talks about us.… So to say, we are used to being exploited’ (original in French: ‘*Bien sûr qu’on est en première ligne, mais personne ne parle de nous.… J’ai envie de te dire, on a l’habitude de se faire exploiter*’).

*There is a growing sense that the post‐quarantine economy will see major tech corporations taking advantage of this new rhetoric of providing ‘essential’ services, the nature of which remains to be proven, as highlighted in Annalisa and Mathilde’s text, with Parisian riders putting their health at risk to deliver ice cream to middle‐class consumers in lockdown. This case opens up further reflection on production and its relationship with repair work*.

## 5 REPAIRING PRODUCTION

**Valeria Graziano and Kim Trogal:** The working conditions of the riders, along with others such as the Amazon warehouse workers protesting over unsafe working conditions (Dzieza, 2020), reflect a further bifurcation and increasing inequalities of the labour that services the domestic realm. This is a realm that in physical isolation has become an intensified site of production, reproduction and consumption. Two different subject positions emerging from this condition are captured well by Ian Alan Paul, who speaks of the ‘domesticated/connected subject, who in being confined to their home is pushed to invent new ways to reconnect to and participate in a virtualized economy’ and ‘the mobile/disposable subject that serves as the circulatory system of the pandemic’ on which the former relies. It is the mobile‐disposable subject who ‘becomes increasingly vulnerable and precarious as it is compelled to move at ever greater velocities’ that is typified by the Deliveroo drivers above (Paul, 2020). This confirms our own work on how technologies intensify labour practices and forms of organization (Graziano & Trogal, forthcoming).

As Naomi Klein put it, rather than gaining momentum for a Green New Deal, the aftermath of the pandemic risks fuelling the composition of what she calls a ‘Screen New Deal’, where techno‐solutionist approaches are presented as ‘benevolent’ interventions in civic life, which are in fact turned into what Lauren Smiley (2020) effectively called a ‘shut‐in economy’. Klein (2020) writes:

*This is a future in which, for the privileged, almost everything is home delivered, either virtually via streaming and cloud technology, or physically via driverless vehicle or drone, then screen ‘shared’ on a mediated platform. It’s a future that employs far fewer teachers, doctors, and drivers. It accepts no cash or credit cards (under guise of virus control) and has skeletal mass transit and far less live art. It’s a future that claims to be run on ‘artificial intelligence’ but is actually held together by tens of millions of anonymous workers tucked away in warehouses, data centers, content moderation mills, electronic sweatshops, lithium mines, industrial farms, meat‐processing plants, and prisons, where they are left unprotected from disease and hyperexploitation. It’s a future in which our every move, our every word, our every relationship is trackable, traceable, and data‐mineable by unprecedented collaborations between government and tech giants*.

What seems to be emerging is a scenario in which the foregrounding of the needs of a ‘care society’ — put forward, alongside other initiatives, via the #CareIncomeNow campaign supported by the Global Women’s Strike (2020) — might be vulnerable to being weaponized as a new justification for public resources being diverted from maintaining, repairing and strengthening existing public infrastructures of social reproduction, towards the coffers of the major players of ‘platform capitalism’ (Srnicek, 2017). Telehealth, remote learning and broadband services are being presented as inevitable by the likes of Bill Gates and former Google CEO Eric Schmidt in their pitches to politicians as they go about securing large sums of public money.

In the meantime, governments at every level are at their weakest ever since the Great Recession, as they are faced with lower revenues from general taxation and the need to face an increase in spending to meet the healthcare needs of their citizens as well as the living needs of new masses of unemployed populations. Countries in the so‐called global South have been the worse hit, if not by the virus itself, by impoverishment, starvation and dislocation. In a world first, Lebanon announced it will default on its debt, while many other countries — such as Pakistan and Argentina — find it difficult to negotiate with the likes of the IMF (International Monetary Fund), which persists in a politics of austerity and privatization measures in exchange for loans.

One of the aspects of the globalized economy that the crisis has also been highlighting is the fragility of long haul, just‐in‐time, delocalized production chains. While these breakdowns have foregrounded the fragility of global supply chains, they might also open up a space for rethinking (and renegotiating) not only the organization of production (including its social reproductive aspects), but crucially, also its purpose vis‐à‐vis a viable and progressive politics of social care.

### 5.1 Production for social care?

One initiative that speaks to such concerns is the recent protest initiated by workers at General Electric (GE) to oppose the company’s plans of laying off nearly 2600 workers working for its domestic aviation division (around 10 per cent of its workforce). On 23 March 2020, workers in Boston and Lynn, Massachusetts staged a silent protest, standing metres apart in compliance with health recommendations. This was not just a protest asking for the preservation of jobs. The workers’ union, the Industrial Division of Communication Workers of America (IUE‐CWA), put forward a more concrete demand of converting the production line from building aircraft engines to much needed ventilators, a demand that they also put to President Trump via an online petition (IUE‐CWA, 2020). While GE is already the United States’ largest manufacturer of ventilators through its Healthcare Division, the production normally undertaken at the Lynn factory is ‘considered essential by the Department of Homeland Security work, as it provides “mission‐critical” equipment to the military’ (Cote, 2020).

The initiative of Lynn’s GE workers links in very concrete ways to people’s need to maintain their livelihoods in the time of crisis — a livelihood that cannot be only understood in terms of salary or income — but as a more expansive need that must be met socially and via the purposes of production more generally.

A similar quest for putting one’s productive capacities at the service of fighting the pandemic has also been seen in many smaller social enterprises such as maker spaces. A paradigmatic example of this diffused phenomenon has taken place in Brescia, Italy, as the local Chiari Hospital faced an emergency within an emergency, when the medical staff realized that the supply of valves necessary for the functioning of a resuscitation tool was running out and that the manufacturer had run out of spare parts due to the high demand. A local 3D printing company, involved by a Milanese fablab, was contacted and they were able to produce a copy of the valve in less than six hours. However, the manufacturer has threatened to sue over intellectual property infringement. Moreover, producing this piece of equipment could also generate further legal troubles for the makers, as equipment classified for medical use has to be officially certified as safe by the health authorities before being put into use, a certification process that takes time and money to achieve (Bria, Cangiano, Fragnito, Graziano, & Romano, 2019; Sher, 2020).

The initiative of GE’s Aviation workers and the Italian 3D printing highlight the potential capacity for at least some factories to convert their production purposes to meet diverse social care needs, including health conceived as a common, and not individual, good. A whole range of industrial manufacturers have been repurposing their facilities to produce goods and equipment to meet the pandemic. Further examples from the UK include gin distilleries producing hand sanitizer, drinks manufacturers producing face masks and donating them to local authorities, car manufacturers producing protective visors and gowns, and many more (Williamson, 2020).

This re‐conversion of production represents an important terrain where different political trajectories are present, and yet they are manifest in an entangled form. The radicality of the pandemic might thus be reclaimed as a temporality for altering the nexus between production, sustainability and social needs. The conversion of facilities to meet an immediate need might, importantly, secure financial survival for those workers. But it simultaneously introduces an opportunity to question the underpinnings of production itself, such as who owns and profits from this production, who decides what is produced, and for whom, and with what effects on the broader environment? As blueprints for how to move forward in this sense are being developed and fought over by the international feminist movement and the climate justice movement — whose goals are today intertwined more than ever — we want to turn to a past example that articulated similar demands for a deep, integrated ‘repairing’ of the purposes of production. This history, bearing similarities to the more recent reaction of the workers at GE, is the Lucas Plan in the UK, conceived by workers of the then publicly owned Lucas Aerospace in 1976.

### 5.2 The right to useful production: The Lucas Plan

When faced with large‐scale job losses through restructuring, driven by the threat of automation and increased global competition, the workers at Lucas Aerospace formed a ‘Combine’^9^ to develop their own alternative corporate plan. The plan set out how their organization could diversify production to move away from the manufacture of arms and military equipment towards, in their words, more ‘socially useful production’ (Salisbury, n.d.). The workers were driven not only by the threat to their own livelihoods, but by an overriding concern in *what* was being produced, and the purposes of industrial production in society. To re‐orient their industry to what they named ‘socially useful production’ meant: questioning the needs products meet (promoting health, welfare); developing technologies that support the development of workers’ skills and knowledge, and which can be controlled by the worker; and designing with repair and re‐use in mind, working against built‐in‐obsolescence (Smith, 2014).

The workers developed over 150 product proposals for a range of sectors including items for medical uses (such as kidney dialysis machines), transportation, the energy sector and more. Aiming to address the energy crisis of the time, their alternative plan included a range of new ecological technologies for heating and powering local homes and communities, such as heat pumps, solar cells, wind turbines and batteries. As researcher Adrian Smith (2014) noted, ‘Even where the technological artefacts were essentially the same … it was *their differentiated relations with production, use and ways of living that proved contentious*.’ Not only did the combine develop practical, useful and ecological products, designed to be affordable and accessible, they also significantly re‐planned the organization to be democratically managed, and re‐planned the role of ‘productive’ industry and business in a locality. The combine’s products should therefore be seen in the context of the democratic and pedagogical processes that accompanied them. The Lucas Plan here points to not only re‐orienting industrial productive capacities, as the call for ‘Green Infrastructure’ and ‘Green Industries’ suggests, but in doing so the need to develop processes that are directed to supporting social reproduction.

### 5.3 Repairing production

While the Lucas Plan was never realized, finding a lack of ‘political will’ in both the UK’s governing Labour Party of the time and a lack of support from the union, it nevertheless represents a significant intervention in the attempt to ‘repair’ and re‐purpose production.

As the COVID‐19 pandemic continues to roll out statistics representing death sentences for the most vulnerable — not just because of the virus in itself, but through impoverishment, malnutrition and lack of access to medical care — the right to a useful production, democratically determined and placed in the hands of workers and those impacted by its broader effects can represent a powerful point of entry into questions of social and environmental sustainability.

Turning to our own places of production, the higher education institution, its growing debt crisis and detrimental and eroding effects of marketization on education and research, the pandemic has also been pointed to as a moment to rethink and repair our own production. News about the unequal impact of the quarantine on female and male academics came as no surprise. In May, an enquiry by *The Guardian* highlighted how the number of article submissions to academic journals from women had dropped dramatically since April, as many reported struggling with childcare, domestic labour and other care duties towards their students. At the same time, submissions by male academics went up by 50 per cent, as reported by some journals (Fazackerley, 2020).

With the financial impacts of the pandemic now being keenly felt in institutions, the coming redundancies and further precarization of staff has prompted the urgent questioning of institutions and highlighted the need for political strategies in response. UK scholars have turned to the Lucas Plan to reconsider not only the organization of universities, but to argue that educators and researchers should re‐question the foundations of what it is we produce and why. They point towards a ‘right to useful production’, demanding the right to democratically self‐organize our institutions, and to ‘press for the right to work on education which is socially useful and helps to solve problems of educational and social inequality rather than creating them’ (Gamsu, 2020).

*In the following piece, Janet Johansson takes another approach in her reflection on the notion of repair from a micro‐perspective of self‐management. This piece turns the argument around and foregrounds care and love in the current striving for repair. With the Buddhist philosophy of Impermanence, and by drawing on Heraclitus’s Flux Theory, she critiques the obsession of normalcy and regularity in general, and the refusal of change in time of crisis in particular. She confronts the fear of change and our fixed vision on sameness and points out that the neglect of the fragility of the most vulnerable people such as the elderly and the chronically ill is the consequence of this fixation in this crisis. With this piece, she calls for profound reflections on normalcy and the acceptance of impermanence as nature and normal. We all need to change so that we can care. By accepting ‘change’ we counteract egocentrism and allow care of the wellbeing of others to be the motivation of the repair work. To care is to be always ready to embrace a different look, voice or situation. To love is to accept vulnerability, is the choice to connect, to break our clinging to sameness and safety, and to find ourselves in the other, and, most importantly, to care about the different Others*.

## 6 NORMALCY VERSUS IMPERMANENCE: A PERSPECTIVE OF CHANGE IN ‘SELF‐MANAGEMENT’ IN THE TIME OF CRISIS

**Janet Johansson:** It has become my morning routine since the virus outbreak in early March in Sweden: every morning, I look out of the window to observe how much the daily life of people has changed. The scenery outside is just like the day before. Parents are walking with their kids to the nearby school. By the waterside, people are out for their morning run or walk, passing by each other through a narrow, temporary passage which is built for the construction work on the sidewalk. Workers have begun their roadworks; the noise from the heavy machines reverberates in the streets and between houses and streets. Everything is the same as the day before. This daily observation makes me rethink about the notion of change, normalcy and regularity.

People inevitably cling to the sense of normalcy and refuse abnormality as if this is the only way of our existence. The urge — to create a normalcy to maintain an unspoken agreement of daily routines, to preserve a set of silently respected rules, which may not, under any circumstances, be violated — functions as a strategy of physical and mental survival. And a sense of pride is deeply rooted in our abilities in maintaining the normalcy and regularity in time of crisis, too. From daily dialogues with friends, colleagues, neighbours and narratives on social media, I capture the expressions of pride. People proclaim that they manage to preserve their lifestyles to the upmost level of normality, such as going to restaurants, parties and visiting friends, as if all denotations of the pandemic including uncertainty, change, suffering, adjustment are being eliminated together with the virus itself when normalcy is maintained.

In English lexicography, ‘normal’, which is defined as ‘constituting, conforming to, not deviating or different from, the common type or standard, regular, usual’ only appeared in 1840 (Davis, 2013, p. 3). The word ‘norm’, ‘normality’ and ‘normalcy’ entered the English language no earlier than 1840 (Davis, 2013). Yet, the insistence of ‘normality’ as ‘good’, ‘healthy’ and integral is persistent. It is appalling to see that the level of refusal of abnormality and irregularity is so profound that people even overlook the fatalities of the pandemic.

In a pandemic, changes are inevitable. The invisible, fatal substance which takes lives is in the air, on the surface of objects, in the water and between each individual. The wheels of the gigantic economic machine that we have vigorously built have slowed down and run off track. Fear is pervasive. The way we normally act, socialize, interact, love, celebrate and mourn will have to change. However, we are obsessed with the notion of safety; and we have learnt that safety lies with the sameness, regularity and normality (e.g., hooks, 2001, p. 93). Difference, abnormality, irregularity and impermanence, on the contrary, are perceived as more dangerous than the fatality of the virus. As hooks (2001) argued, cultural domination relies ‘on the cultivation of fear as a way to ensure obedience’ (p. 93). Thus, whoever obtains the authority to define the content of fear, wins the power to shape perpetual desires, normalcy and regularity as the culture we live in and are led by. People have been told that the real threat of a pandemic is not a deadly virus, but the disruption of regular routines and slowing down of the economy and consumption. As a consequence, many — especially those who are young and healthy, who have been told that they are risk‐free of the infection — strive for maintaining normalcy to preserve life as usual. However, the overrated notion of normality obscures our attention from the lives of the vulnerable and disconnects us from those who cannot afford to fight this war alone.

Have we, as human beings, always persistently refused to face impermanency or is it something we have learned or being told to do? Originally, the ancient Greeks linked the concept of the ‘normal’ with that of the ‘natural’. Nature is not regular; the irregular is also natural and therefore ‘normal’ (Ickstadt, 2000, pp. 6–7). The meanings of ‘normal’, however, have evolved along with societal ideals of ‘good’, ‘healthy’ or ‘perfection’. In this pandemic, some people accept the idea of temporary irregularity, they adapt to changing conditions, endure the fear of abnormity, with the hope to quickly repair, recover, retrieve and restore normalcy. Others refuse to disrupt regular routines. For those people, change, irregularity and abnormity are pathologies and therefore cause despair, distress and desperation.

Indeed, living, like loving, involves the concept of safety, permanence and regularity. However, living, also like loving, is a natural process that is connected to change, broken, impermanent and even suffering, which eventually leads to learning and growing. To love, as Lewis (1960) put forward, is to be vulnerable. To love, for hooks (2001), is the choice to connect, to break our clinging to sameness and safety, to find ourselves in the other and, most importantly, to care. Whereas feminist theorists emphasize the recognition and empathy of the vulnerability of others as the ground of ethics of care because persons are relational and societies are potentially caring morally and epistemologically (Held, 2005; Tronto, 1993). Therefore, to sustain normalcy is inseparable from the connection with and the care for others.

Moreover, Heraclitus, in his flux theory, spoke about the fluid nature of things: ‘There is no static being no unchanging substratum. Change, movement, is the Lord of the Universe. Everything is in a state of becoming, of continual flux (Panta Rhei)’ (in Thera, 1981, p. 8). This notion coincides with the Buddhist philosophy of Impermanence, which opens another perspective on the paradoxical concepts between normalcy, irregularity and abnormality. With this, Buddhism problematizes the essentialist concept of the so‐called being and existence (Thera, 1981). In Buddhism, change or Impermanence is the essential characteristic of all phenomenal existence. With the theory of Impermanence, all is fleeting; the crisp air, the affection of love, the rays of sunshine, the beauty of flowers, the glory of stars, they all rise, deteriorate, vanish and then recuperate. Hence, Impermanence corresponds to normalcy, and abnormality is equivalent to regularity. To embrace Impermanence, to alter tracks, to reflect upon our lives that have been completely consumed in the economic vortex, and to change habits, rituals, as well as routines are perhaps the true premises for ‘good’ and ‘healthy’, which, in turn, may open a space for ethical care and love for those who are vulnerable and dependent on changes of routines to survive. Perhaps the morale of the pandemic is to rouse the ancient wisdom to live again, be it once spoken by Heraclitus or the Buddha.

This is a new day. I see a small metal boat quickly sailing by. A father and his two children are on board. I have seen them before. This is their morning routine. They quickly but orderly get onshore, tie the boat and then the children say their goodbyes before rushing to schools. Seeing them today is different. Their slight but usual stress reminds me of normalcy. Yet, I say to myself silently that I will use every precious moment of this day, to change. The desire for change has intrigued the thoughts of overturning the ‘norms’ that have been ruling habits, routines and perpetuating our fear and desires for too long. The change I am looking for is not merely the day‐to‐day adaptiveness which ensures a temporary state of stability before repair and restoration. Instead, it is the awakening to the recognition that Impermanence is the new form of ‘normalcy’ and life is flux. Our selves need a profound change, a change that involves the new vision of our existence in connection with others and Nature. May this change begin today!

## 7 COLLECTIVE EPILOGUE: WALKING FEMINIST FRONTIERS

This work began with Silvia’s reflection on the notion of repair work during COVID‐19 and her invitation for collaborative writing. We are seven women from different nations, of different ethnicities and in different phases of our lives. Unanimously, we all took ‘care’ as the central theme in our *reflections and imaginaries* of repair work. Following the invitation and with different focuses and perspectives, we translate the efforts we have made in responding to the crisis, the ways we have faced up to the breakdown of normalcy, actions we take to repair the social order into opportunities for critical‐reflexive thinking, learning, growing and engineering heterogeneity, as well as challenging the *status quo*.

As different as the authors’ choice of perspectives, the focus and critique is on challenging the lack of care in conventional forms of organizing by calling for the development of ethico‐political relations in decision‐making processes and affirmative politics for change (Hancock, 2008; Pullen & Rhodes, 2014, 2015a, 2015b; Pullen, Rhodes, & Thanem, 2017; Vachhani & Pullen, 2019), more specifically, by directing attention to the affective aspects of intersubjective relations (Benozzo et al., 2019; Fotaki, Kenny, & Vachhani, 2017; Thanem & Wallenberg, 2015) and attending to individual and collective vulnerability and interdependency (e.g., Tyler, 2018).

Tronto (1993) pushes the ethic of care into political discourse by arguing that we must understand care as a social practice rather than a disposition that is ‘easy to sentimentalise and privatise’ (p. 118). This notion transforms the status of care and of women. Feminist writing thus presents an ethic of care as social practices emphasizing the concrete needs of people and valuing the notion of growth (Gilligan, 1982; Ruddick, 1980). Interestingly, in this work, by critiquing the invisible female gender in media exposure of care work, arguing for the missing voices of delivery riders in Paris, urging ethical thinking about care in the decision‐making of productions and by encouraging a new rationale around the concept of normalcy, we eventually unite in terms of concern and theorization of care through writing one collaborative text on the notion of *repair* differently.

Writing together around COVID‐19 and beyond is a privilege that allows us to take a breath and reflect on events without distancing ourselves from our embodied experiences, to give voice to Others because the Others have a voice that must be heard, to denounce multiple invisibilities as well as rhetoric visibilities. Writing together as women, feminists, rather than academics, is a practice of resistance and resilience that in our case becomes *a conversation about mending the social texture* dramatically broken by the outbreak, *having in mind a feminist ethos of care*, to use Silvia’s words.

‘Mending’ (from the Old French ‘amender’, that is, ‘correct, set right, make better, improve’) and ‘repair’ (from Latin ‘re‐’, that is, ‘again’ and ‘parare’, that is, ‘make ready, prepare’) have in common the meaning of ‘putting back in order’, which generally corresponds to people’s main hope during a pandemic: going back to normality. But, as Janet questions, is this obsession of ‘going back’ reasonable since the insistence of normalcy and regularity have caused the negligence of the needs of the vulnerable and cost the lives of the unhealthy? *In a pandemic, changes are* inevitable, she points out, and if there is a moral for the pandemic, it is *to embrace Impermanence* and be ready for changes and for care, as well as to connect ourselves with the needs of others.

Walking the empty space of a page to fill it diffractively by experiencing our own being and becoming together‐with, means to us moving forward instead of repairing the old order as it was before the breakdown and asking ourselves — even before asking anyone else — ‘what radical possibilities for change are there in practice and theory?’ (Pullen, Lewis, & Ozkazanc‐Pan, 2019, p. 6). This question resonates with Arundhati Roy’s (2020) recent words:

*Historically, pandemics have forced humans to break with the past and imagine their world anew. This one is no different. It is a portal, a gateway between one world and the next. We can choose to walk through it, dragging the carcasses of our prejudice and hatred, our avarice, our data banks and dead ideas, our dead rivers and smoky skies behind us. Or we can walk through lightly, with little luggage, ready to imagine another world. And ready to fight for it*.

We then take a stand and say, with Silvia, that we *need to prevent the future from becoming the past. We are not going back to the past; our society has already changed and there is a need to cope with innovation and repairing practices that do not reproduce the past*. The breakdown is actually multiple in its meanings and implications for our western epistemological assumptions of control and domain. What COVID‐19 is foregrounding are the *structural invisibilization dynamics based on class and race* denounced by Annalisa and Mathilde, as well as the fragility of our globalized organizational — productive and reproductive — systems. However, this crisis performs a space *for rethinking (and renegotiating)* these systems, as Valeria and Kim point out. *History repeats itself*, Michela notes, but, by gratefully reading the many contributions that have preceded our own in this section of *Feminist Frontiers*, we reclaim the idea that the future can be otherwise and a renewed feminist practice — whatever it means everyone individually — can contribute to starting a new page that can be filled in together‐again‐but‐differently.

## DECLARATION OF CONFLICTING INTERESTS

The authors declared no potential conflicts of interests with respect to the authorship and/or publication of this article.

## ENDNOTES

1 ‘Your Stories: Through the Eyes of a COVID Nurse who Tested Positive’, see https://www.healthcareitnews.com/news/your-stories-through-eyes-covid-nurse-who-tested-positive

2 Intensive care unit nurse talks about how COVID‐19 has changed her life, see https://abcnews.go.com/Health/icu-nurse-life-changed-battle-zone/story?id=70207389

3 This excerpt is part of the nurse’s story ‘*Infermiera in prima linea racconta le condizioni dei malati di Covid‐a9: e’ un calvario*’, see https://www.quotidianopiemontese.it/2020/05/01/infermiera-in-prima-linea-racconta-le-condizioni-dei-malati-di-covid-19-e-un-calvario/?fbclid=IwAR33pbW90mhut66DWc7L_jLu_W1edl4-x-dlFU9z5oydLUe4HpsRJnf4UVk

4 I wish to thank Augusta Nicoli and the Social Innovation Area of the Agenzia Santaria e Sociale della Regione Emilia‐Romagna for giving me access to the project of innovation after COVID‐19 that they promoted, see https://assr.regione.emilia-romagna.it/attivita/innovazione-sociale/oss-pratiche-inn-covid/intro

5 The analysis giving rise to this contribution was conducted in the frame of the SHARE research project. The project SHARE — Seizing the Hybrid Areas of work by Re‐presenting self‐Employment — has been funded from the European Research Council (ERC) under the European Union’s Horizon 2020 research and innovation programme (Grant Agreement No. 715950).

6
https://twitter.com/i/status/1245366601299496960

7
https://twitter.com/_CLAP75/status/1245378253742845957?s=20

8 The following quotes are excerpts from: (i) a video report produced by Brut. 21 March, following Hamza during a work evening (https://www.brut.media/fr/news/livreur-il-aurait-prefere-etre-confine-hamza-temoigne-40ddbba0-4936-474b-b403-186c58db57a0); (ii) a radio programme entitled *Les pieds sur terre* broadcast on 10 April on France Culture, based on the testimony of Vincent (https://www.franceculture.fr/emissions/les-pieds-sur-terre/journal-de-non-confinement-une-caissiere-et-un-livreur).

9 The combine was a representative body of staff and manual worker unions on all 15 sites throughout the UK. It had been established by the shop stewards to enable the workforce to maintain a coherent and unified voice when responding to management’s corporate view on wages, pensions, manning levels and other such issues. The Story of the Lucas Plan, see https://lucasplan.org.uk/story-of-the-lucas-plan/
